# Supplementary material for: Near-atomic resolution structures of interdigitated nucleosome fibres
Source: Nat Commun. 2020 Sep 21;11:4747. doi: 10.1038/s41467-020-18533-2 (PMC7505979; doi:10.1038/s41467-020-18533-2)
Supplement: Supplementary file 1 — Supplementary Information [file 41467_2020_18533_MOESM1_ESM.pdf]

Near Atomic Resolution Structures of Interdigitated Nucleosome Fibres

SUPPLEMENTARY INFORMATION

Zenita Adhireksan, Deepti Sharma, Phoi Leng Lee & Curt A. Davey

CONTENTS

6 Figures: pages 2-9

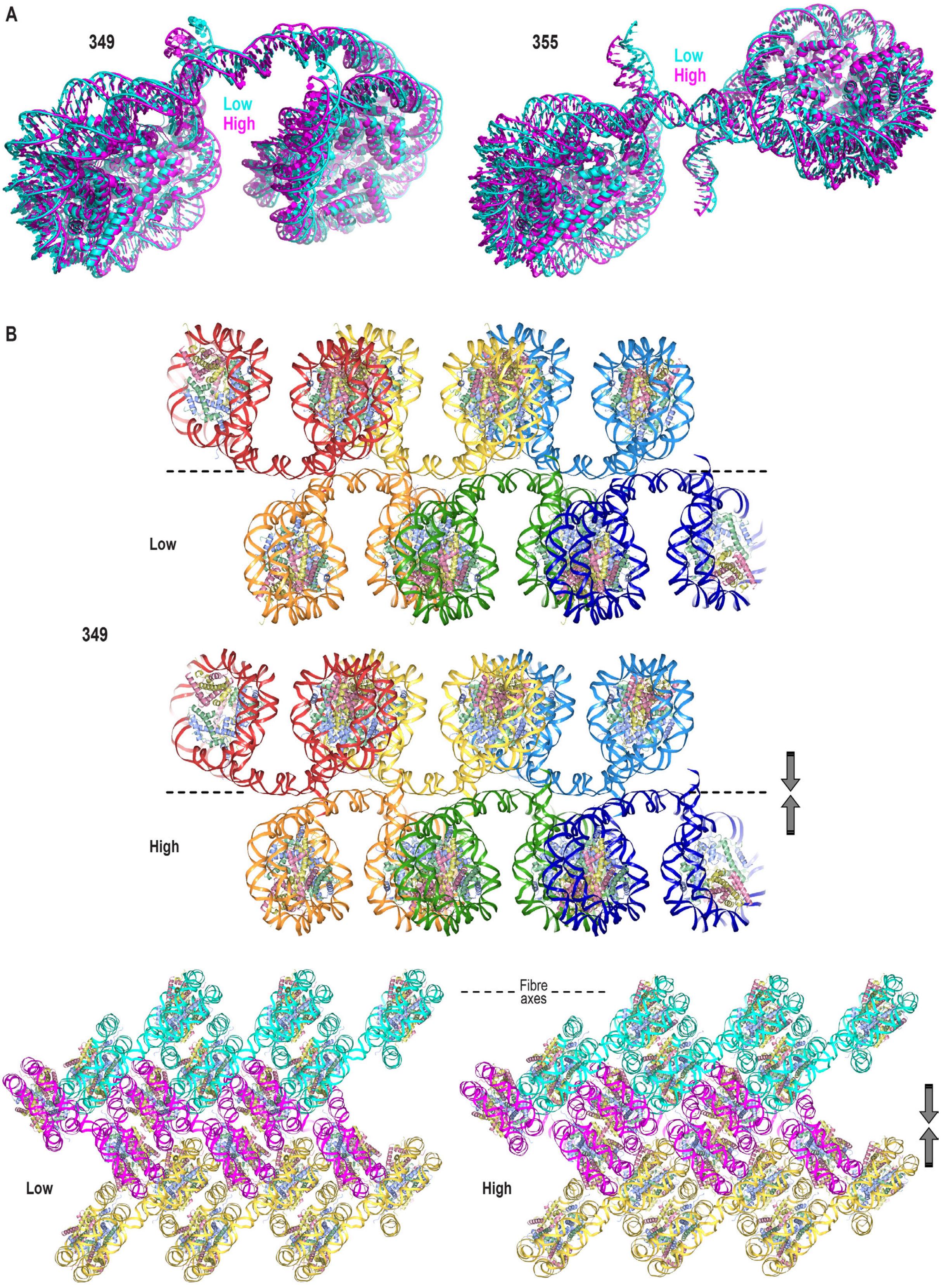
A
349
Low
High
355
Low
High
B
Low
349
High
Fibre
axes
Low
High

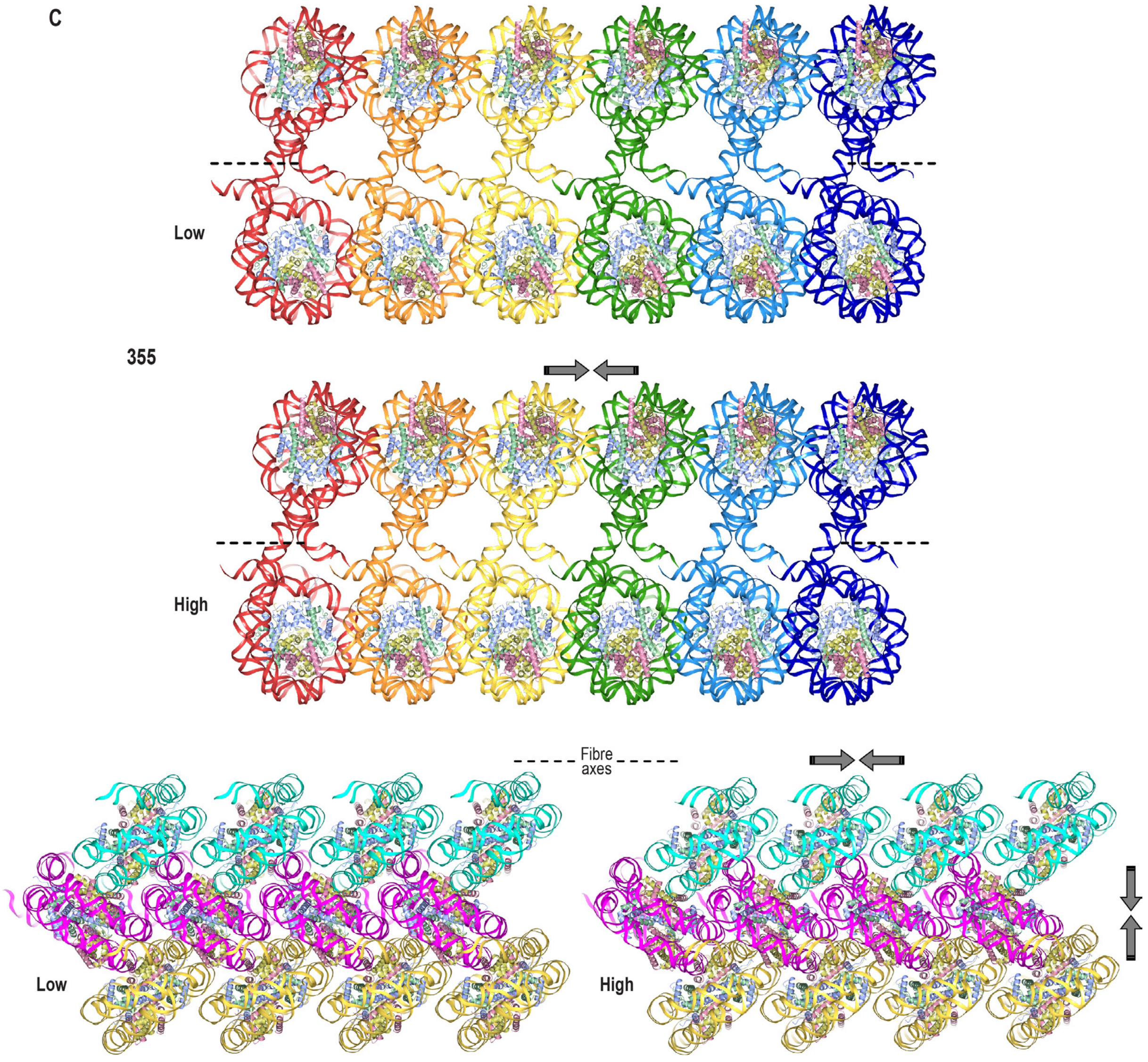

Supplementary Figure 1. Nucleosome fibre packing density increases with dehydration. In order to optimize diffraction quality, data sets were collected for 349 and 355 crystals at both low and high cryoprotectant concentrations. (**a**) Comparison of the dinucleosome asymmetric units between low and high cryoprotectant conditions (dinucleosome structures least-squares superimposed). (**b**, **c**) Comparison of fibre structures and fibre packing between the low and high cryoprotectant conditions. The dehydrating effect from elevated cryoprotectant concentration is apparent in a contraction of the lattice, or unit cell. Although the dinucleosome repeats display significant structural differences between the two hydration levels from conformational changes in the linker DNA (r.m.s.d. of superimposed

dinucleosomes is 3.01 and 5.05 Å for the 349 and 355 systems, respectively; **a**), the overall structural nature of the individual fibres and their interdigitation remains the same (**b**, **c**). The effect of dehydration is largely to compress the lattice orthogonal to the nucleosome fibre axes.

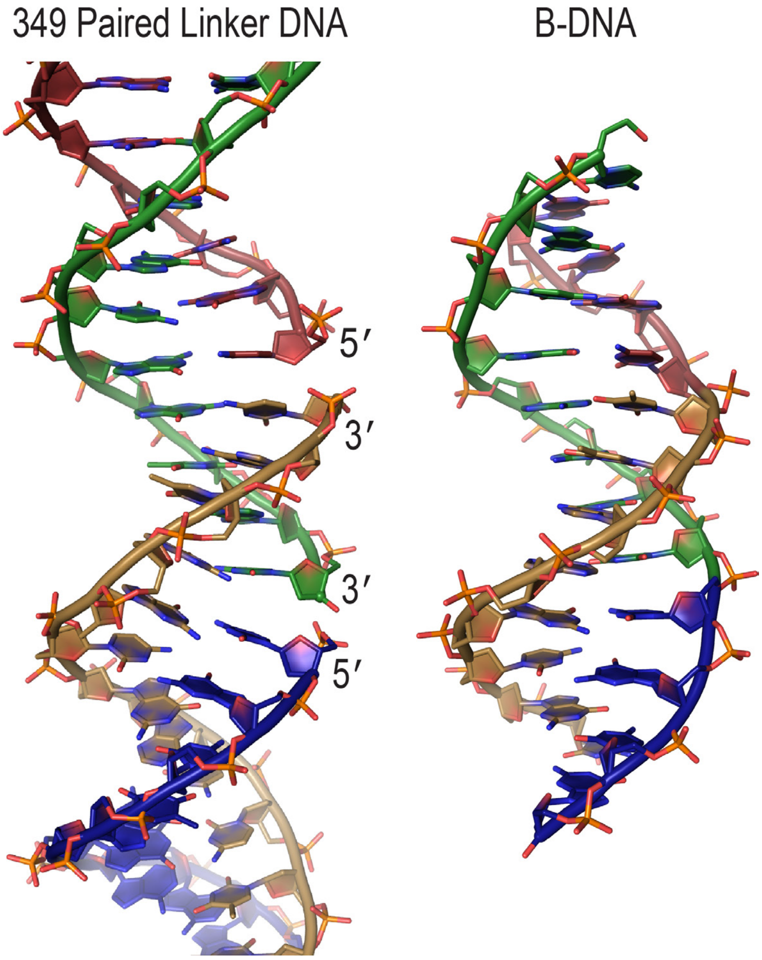

Supplementary Figure 2. Continuity of the double helix from annealing of the cohesive termini in the paired linker DNA sections. One of the paired linker DNA sections from the 349 fibre is shown is comparison with B-form DNA (pdb code 1BNA). The carbon backbones of the DNA strands are coloured sand/dark blue and green/dark red, respectively, for the two paired dinucleosomes. To emphasize their similarity, the same colouring scheme is used for the respective regions of the B-DNA structure.

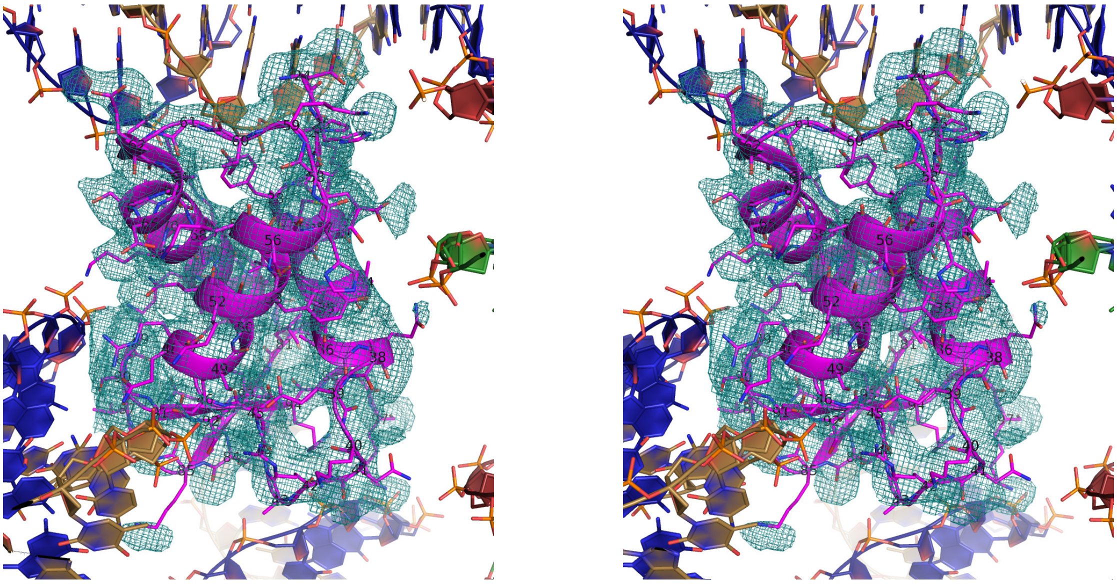

Supplementary Figure 3. Experimental electron density corresponding to on-dyad linker histone (H1.0) binding in the 349 fibre crystals, shown in stereo view. An $F_O - F_C$ omit electron density map (teal; contoured at 2σ; linker histone atoms omitted from the model) in the vicinity of the omitted atoms is superimposed onto the refined model.

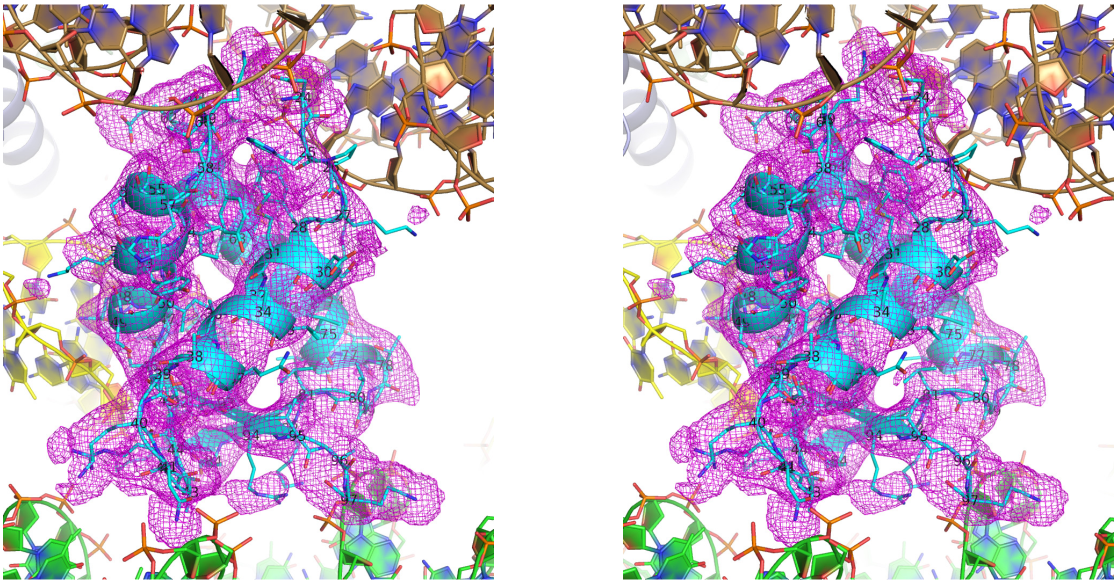

Supplementary Figure 4. Experimental electron density corresponding to non-dyad linker histone (H1.0) binding in the 349 fibre crystals, shown in stereo view. An $F_O - F_C$ omit electron density map (magenta; contoured at 2σ; linker histone atoms omitted from the model) in the vicinity of the omitted atoms is superimposed onto the refined model.

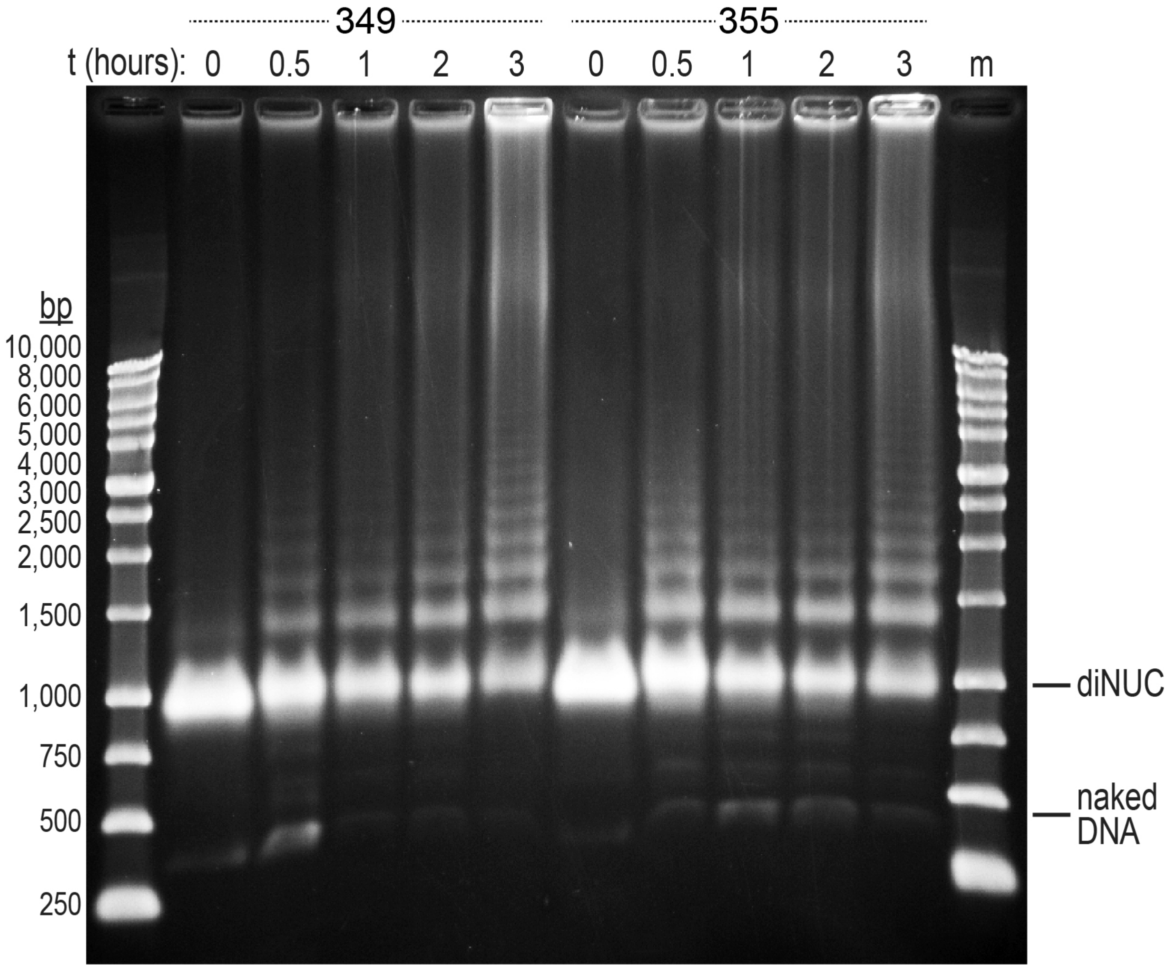

Supplementary Figure 5. Long fibres of the 349 and 355 dinucleosomes form in solution. 349 and 355 dinucleosome (diNUC) were subjected to treatment with ligase for varying times, followed by agarose gel electrophoresis and ethidium bromide staining. DNA fragment molecular weight marker (m) is run at the sides.

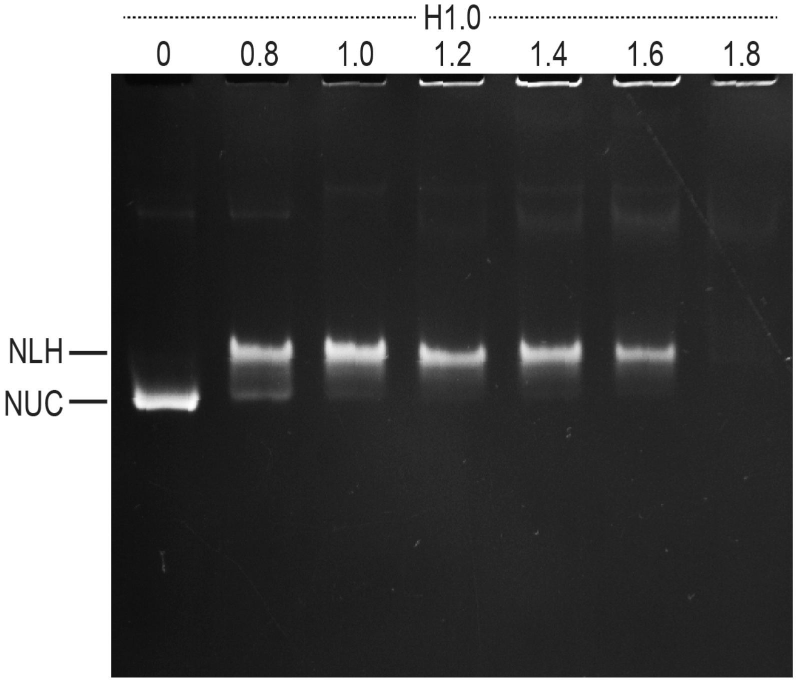

Supplementary Figure 6. Electrophoretic mobility shift assay of linker histone binding to nucleosome. Linker histone (H1.0) was incubated with nucleosome (NUC) at different H1.0:NUC molar stoichiometry and subjected to native PAGE analysis. Nucleosome-linker histone assembly (NLH) is also known as chromatosome.
